# Molecular and cellular pathways contributing to brain aging

**DOI:** 10.1186/s12993-021-00179-9

**Published:** 2021-06-12

**Authors:** Aliabbas Zia, Ali Mohammad Pourbagher-Shahri, Tahereh Farkhondeh, Saeed Samarghandian

**Affiliations:** 1 Department of Biochemistry, Institute of Biochemistry and Biophysics (IBB), University of Tehran, Tehran, Iran; 2 Medical Toxicology and Drug Abuse Research Center (MTDRC), Birjand University of Medical Sciences (BUMS), 9717853577 Birjand, Iran; 3 Cardiovascular Diseases Research Center, Birjand University of Medical Sciences, Birjand, Iran; 4 Faculty of Pharmacy, Birjand University of Medical Sciences, Birjand, Iran; 5 Noncommunicable Diseases Research Center, Neyshabur University of Medical Sciences, Neyshabur, Iran

**Keywords:** Brain, Aging, Oxidative stress, Inflammation

## Abstract

Aging is the leading risk factor for several age-associated diseases such as neurodegenerative diseases. Understanding the biology of aging mechanisms is essential to the pursuit of brain health. In this regard, brain aging is defined by a gradual decrease in neurophysiological functions, impaired adaptive neuroplasticity, dysregulation of neuronal Ca^2+^ homeostasis, neuroinflammation, and oxidatively modified molecules and organelles. Numerous pathways lead to brain aging, including increased oxidative stress, inflammation, disturbances in energy metabolism such as deregulated autophagy, mitochondrial dysfunction, and IGF-1, mTOR, ROS, AMPK, SIRTs, and p53 as central modulators of the metabolic control, connecting aging to the pathways, which lead to neurodegenerative disorders. Also, calorie restriction (CR), physical exercise, and mental activities can extend lifespan and increase nervous system resistance to age-associated neurodegenerative diseases. The neuroprotective effect of CR involves increased protection against ROS generation, maintenance of cellular Ca^2+^ homeostasis, and inhibition of apoptosis. The recent evidence about the modem molecular and cellular methods in neurobiology to brain aging is exhibiting a significant potential in brain cells for adaptation to aging and resistance to neurodegenerative disorders.

## Introduction

Aging is a complex and multifactorial phenomenon characterized by a gradual decrement in physiological actions and behavioral capacity. Aging happens at molecular, cellular, and histological levels in different organs, particularly at the central nervous system (CNS) and particularly the brain [1–3]. Advancing of age alters the biological, chemical, and physical functions of neurons leading to memory impairment, altered behaviors, loss of cognition functions, dementia, and impaired immune responses. Also, aging is one of the main risk factors for common neurodegenerative disorders such as Parkinson's disease (PD), amyotrophic lateral sclerosis (ALS), Alzheimer's disease (AD), and stroke [4, 5]. Brain aging has been linked with subtle alterations in the structure and function of neurons in specific neuronal circuits rather than only high rates of neuron loss. The compensation mechanism for neuron loss in the aging brain is through expanding dendritic arbors and synaptic connections. The brain in age-associated neurodegenerative diseases loses dendritic arbors and synaptic contacts. It thus cannot compensate for the neuronal loss [6, 7].

Brain aging in the various species of mammals allocates several common characteristics, such as synaptic atrophy, dendritic regression in pyramidal neurons, accumulation of fluorescent pigments, cytoskeletal abnormalities, a decline of striatal dopamine receptors, and reactive microglia and astrocytes [8]. Despite the identification of brain aging characteristics in several neuronal networks of the brain, the exact responsible molecular mechanisms are still unclear.

Studies have suggested several major signaling pathways and molecular targets, including oxidative stress, metabolism dysfunction, deregulated autophagy, telomere shortening, mitochondrial dysfunction, cellular calcium homeostasis, and systemic inflammation modulation of aging and lifespan in a wide range of species expanding from yeast to mammals. Gene expression analysis studies have defined some of these signaling and molecular pathways and led to conserved evolutionary alterations through aging [6, 9, 10]. Further, brain imaging technologies such as magnetic resonance imaging (MRI) and positron emission tomography (PET) are allowing the study of neuronal circuits and cognitive networks in the aging brain [11].

Several environmental factors have been identified to affect brain aging mechanism, including the rate of structural alteration. Calorie restriction (CR) has been shown to delay functional and structural decreases by aging in some animal model organisms such as rodents and monkeys [12, 13]. CR reduces daily calorie intake below the energy requirements while keeping optimal nutrients for health without being malnutritional [14, 15]. It was first shown by McCay et al. in 1935 that male and female rats with CR had increased lifespan while having a retarded growth [16].

Considerable evidence indicates the beneficial effects of physical exercise on healthy aging, even in old age [17–19]. Several epidemiological studies have investigated the effects of physical exercise on cognition in older age. A systematic review of 127 observational studies showed that physical exercise decreased the relative risk of cognition decline with risk or hazard ratios in the range of 0.6 and 1.6 in the general population over 50 years old in economically developed countries [20]. A systematic review of 33,816 non-demented individuals older than 65 years, followed between 1 and 12 years, who were enrolled in 13 prospective studies showed that 3210 individuals developed cognitive decline. Individuals with high levels of physical exercise had reduced cognitive decline with a hazard ratio of 0.62 (95% CI 0.54 to 0.70; pb0.00001), and low to moderate physical exercise reduced the risk of cognitive decline with a hazard ratio of 0.65 (95% CI 0.57 to 0.75; pb0.00001) compared with sedentarism [21].

Physical exercise has been shown to protect against brain aging by inducing angiogenesis and increasing the blood flow to the brain [22–25]. The underlying mechanisms for these effects of physical exercise are thought to be through inducing vascular endothelial growth factor (VEGF) [26, 27]. Animal researches showed that physical exercise initiates neurogenesis, evidenced by increased proliferation and survival of hippocampal cells ranging from young to old age animals [28–31]. Furthermore, physical exercise was shown to induce and maintain LTP. Also, it enhanced short-term memory in the DG by exhibiting hippocampal neurogenesis in animals [28, 32, 33].

Studies from rodents showed that physical exercise could counter age-related cognition impairments by attenuating and even reversing the age-induced decreased neurogenesis [32, 34]. However, one of the limitations for beneficial effects of physical exercise in rodents was the age of animals as very old rodents did not benefit from the physical exercise [35]. It has been shown that physical exercise protects against AD pathology via suppressing amyloid deposition and reducing Ab-dependent neuronal cell death [36–38]. Also, type, frequency, and amount of calorie intake and physical exercise can significantly affect brain health during the lifetime. For instance, aerobic exercise enhances hippocampal volume, while excessive calorie consumption, inadequate physical activities, and obesity accelerate hippocampal atrophy [39].

By increasing dopamine receptors' expression in the striatum, physical exercise combat age-related cognitive decline [40]. In the same concept, physical exercise in the form of treadmill running protected against PD by recovering the dopamine depletion in the striatum of hemiparkinsonian rats [41].

Mental activities can exhibit neuroprotection and neuroplasticity enhancement during aging. It has been shown that treads of mental engagements such as educational level, occupational complexity, or cognitive lifestyle activities can reduce the risk of cognitive impairments [42, 43]. Interestingly, mental activities in individuals with a high-risk genetic background (e.g., apolipoprotein E4 carriers) for the cognitive decline can delay the clinical manifestation of cognitive decline [44–46]. A meta-analysis showed that individuals with high mental activities impose lower cognitive decline risks in the future [47]. Studies have investigated mental activities in the concept of environmental enrichment, which comprises augmented living conditions, including more space, increased social contact, increased cognitive stimulation through toys, wheels, mazes, and more opportunities for physical activity [48].

Several aging theories have been suggested to describe the process of aging and, in particular the brain aging. However, neither of them seems to fill the gap. Hence, we discuss molecular, cellular, and neuro-anatomical changes integrated through brain aging and age-associated neurodegenerative disease. The role of critical metabolic pathways has also been discussed.

## Role of oxidative stress in brain aging

In the 1950s, Harman's free radical theory of aging suggested that reactive oxygen species (ROS) cause oxidative damage in cellular macromolecules, including DNA, proteins, and lipids, leading to decreased biochemical and physiological function through aging [49, 50]. In mammals, ROS origins include xanthine oxidase, NADPH oxidase, nitric oxide synthase (NOS), peroxidase, cyclooxygenase and lipoxygenase, mitochondrial respiration, cytochrome p450s, and endoplasmic reticulum [51, 52]. Free radicals are a subset of ROS species possessing unpaired electrons. These free radicals include peroxyl (RO_2_·), nitric oxide (NO·), hydroxyl radical (HO·), superoxide anion (O^2−^·), and hydroperoxyl (HO_2_·). Other ROS like peroxynitrite (ONOO −), ozone (O_3_), hypochlorous acid (HOCl), and hydrogen peroxide (H2O2) are non-radical derivatives that have role in oxidative stress [53].

The hemostasis of intracellular ROS is mediated via the activity of numerous enzymatic and non-enzymatic systems with the cellular detoxification function, cumulatively called antioxidants.

The nuclear factor erythroid 2-related factor 2 (Nrf-2) is the main transcription factor and also one of the primary regulators of the antioxidant signaling, such as transcription of endogenous antioxidant enzymes including glutathione (GSH), glutathione reductase (GR), glutathione peroxidase (GPx), catalase (CAT), superoxide dismutase (SOD) and heme oxygenase-1 (HO-1). This antioxidant system is collectively the primary defense system that neutralizes ROS generation inside the cells [54, 55].

Mitochondria are considered a significant origin of ROS through aging [56]. Harman, in the 1970s, reviewed his free radical theory after the discovery of the fact that mitochondria can produce water from oxygen at the end of the electron transport chain (ETC), a procedure that, when impaired, lead to a high generation of mitochondrial ROS (mtROS), which is associated with high levels of mitochondrial DNA (mtDNA) mutations and respiratory chain deficiency [57].

In *D. melanogaster*, the signaling function of mtROS produced by the respiratory chain (RC) positively affects lifespan, helping to extend longevity [58]. Knockdown of the SOD2 gene and enhanced mtROS appears to extend lifespan in *C. elegans.* In contrast, up-regulation of catalase enhances oxidative stress resistance and does not extends lifespan [59, 60]. Also, dietary supplements of antioxidants decrease the lifespan of worms [61]. Interestingly, the genetic modification that increased mtROS and ROS-induced injury in mice did not accelerate the progression of aging; however, it promoted the appearance of different age-associated disorders [62]. The reason is that cytosolic ROS and mROS have opposite effects, and cytosolic ROS is more harmful [63]. In detail, the hydrogen peroxide generated with beneficial purposes in mitochondria spreads across the membrane of mitochondria, releases to the cytosol, and functions as an oxidative agent involved in aging [64]. This evidence proposes that ROS effects depend on their concentration and space [63].

The types of ROS in neurons include superoxide anion produced by the respiratory chain and via different oxidases, hydroxyl radical generated via the hydrogen peroxide reaction with Cu^+^ or Fe^2+^, and NO produced in response to increased intracellular levels of Ca^2+^ [65]. Also, in the mitochondria, NO is generated from L-citrulline and L-arginine in a NOS reaction. This enzyme has three different isoforms with various tissue localizations. Endothelial NOS (eNOS) is expressed in the vascular endothelium, cellular Ca^2+^-dependent neuronal NOS (nNOS) is expressed in glial cells (microglial cells, astrocytes, and macrophages) and Ca^2+^-independent inducible NOS (iNOS), which is expressed in a wide range of cells and tissues. NO is involved in several essential procedures within the CNS, like memory formation and the regulation of cerebral blood flow (CBF) to provide a sufficient supply of blood to the brain. Also, it has a pivotal role in modulating the immune system, including the regulation of cytokine generation [66].

During brain aging, enhanced ROS generation and decreased antioxidants result in redox imbalance, causing age-related disorders. In a mouse model of AD, voluntary physical exercise suppressed oxidative stress in the brain, evidenced by reduced oxidative stress biomarkers and down-regulated pro-inflammatory and pro-oxidative mediators [67]. NO-dependent oxidative damage promotes apoptosis in motor neurons. It causes vascular cognitive impairment through the aging of the cerebral cortex. Further, NO-mediated neuronal injury is involved in several neuronal disorders such as PD [68]. ROS can deteriorate cellular function via the modification of proteins and lipids in the brain. The alterations in the composition of phospholipids provide evidence that ROS-induced lipid peroxidation happens in brains involved in CNS dysfunction, like cognitive impairment, in aged humans and animals. Accordingly, enhanced production of malondialdehyde (MDA) in brains has been proposed as a hallmark of aging. It has been reported that the levels of MDA were remarkably increased in the aged rat hippocampus [69]. Also, MDA on electron microscopy forms cap-like linear deposits connected with intracellular neuronal lipofuscin.

In contrast, glial MDA deposits surrounded vacuoles in the CA4 region of the human hippocampus [70]. On the other hand, no remarkable enhancement of MDA-modified proteins was found in the brain of the aged rat, which could be because MDA-modified protein reacts with lipid peroxidation products cross-link with each other [71]. In the cerebral cortex and cerebellum of aged rats, diminished amounts of poly saturated fatty acid (PUFA) and enhanced amounts of monounsaturated fatty acids have been reported [72].

The capability to maintain long-term potentiation (LTP) is commonly used as the main factor of cognitive function. Decreased levels of arachidonic acid (AA) in the aged rat brain have been associated with impairment of LTP. This proposes that oxidative reduction of AA levels might associate with cognitive impairment in rats [73, 74]. It was reported that brains of aged dogs exhibit lipid peroxidation product 4-hydroxynonenal (HNE) accumulation, linked with neurofibrillary tangles (NFTs) and amyloid deposits [75]. Post-synaptic AMPA receptors are implicated in the LTP process [76]. It has been shown that after five weeks of environmental enrichment, LTP and LTD were increased [77]. Upregulation of post-synaptic AMPA receptors and increased LTP-related functions have been shown to occur after environmental enrichment in mice with NMDA defects [78]. However, in mice with upregulated NMDA receptors, environmental enrichment did not affect functions related to LTP [79].

Many investigations exhibited that brain aging was connected with the accumulation of oxidized protein, characterized by elevated carbonyl residues [80, 81]. Also, it was exhibited that levels of protein carbonyl were enhanced in different regions of the brain in various animal models, like the occipital and frontal cortex in aged humans [81], the forebrain in Wistar rats [82], brain homogenates of aged canine models [80] and the cortex in Brown Norway rats [83] and Mongolian gerbils [84]. Following the idea that oxidative stress-mediated enhanced protein carbonyls contribute to dementia detected in some older adults, in the aged learning-impaired rats, only proteins in a 65-KDa band were detected to have high levels of carbonyl residues [85]. Decreased olfaction is one of the common characteristics of aging. Investigations of model organisms like mice exhibit that amounts of protein carbonylation in astrocytes and neurons and protein nitration in blood vessels are enhanced in the olfactory bulb through aging [68].

Decreased antioxidant defense and disability to eliminate oxidatively damaged biomolecules can accelerate the aging of organisms. In confirming, genetic reduction at the levels of SOD2 in *D. melanogaster* and mice results in the accelerated onset of age-related neurological characteristics, such as neuronal DNA damage, motor dysfunction, and neurodegeneration [86, 87]. One of the major antioxidant defense complexes is the heat-shock response (HSR), a cellular response that elevates the number of molecular chaperones to diminish the adverse effects on proteins caused via stressors, oxidative stress, increased temperatures, and heavy metals. Long-term exposure to extreme stress conditions is detrimental. Activation of heat-shock protein (HSP) synthesis can lead to enhanced stress tolerance and cellular protection against neuronal injury [88–90]. In the CNS, HSP synthesis is induced via exposure to cytotoxic drugs, heavy metals or amino acid analogs, and changes in the intracellular redox state [88, 89]. Therefore, the heat-shock response has a crucial role in providing a cytoprotective environment in the different metabolic disruptions, like age-associated neurodegenerative disorders and aging. Also, in aged animals, oxidative modification of proteins leads to protein aggregation, cellular damage, diminished organ function, and ultimately to apoptotic cell death. HSP has a pivotal role in maintaining cells from injury via defense against protein denaturation. Hence, alterations of the activity and expression of HSPs are associated with the denaturation of proteins during aging [91–93].

Similarly, the GSH system is necessary for the cellular detoxification of ROS in brain cells. A compromised GSH system in the brain has been associated with oxidative stress in neurological disorders [94–96]. Interestingly recent evidence shows that GSH has pivotal extracellular functions in the brain. Astrocytes seem to have a crucial role in the metabolism of GSH in the brain since the release of GSH from astrocytes is essential for providing precursors for GSH synthesis in neurons [97]. Between the various brain cells investigated in vitro, only cultured astrocytes produce and release significant GSH and GSSG through oxidative stress [98]. Recent experiments propose a direct association between diminished levels of GSH by oxidative stress and rapid up-regulation of HO-1 in a different range of cells such as human fibroblasts, rat cardiomyocytes, and rat brains [99].

**Fig. 1:**
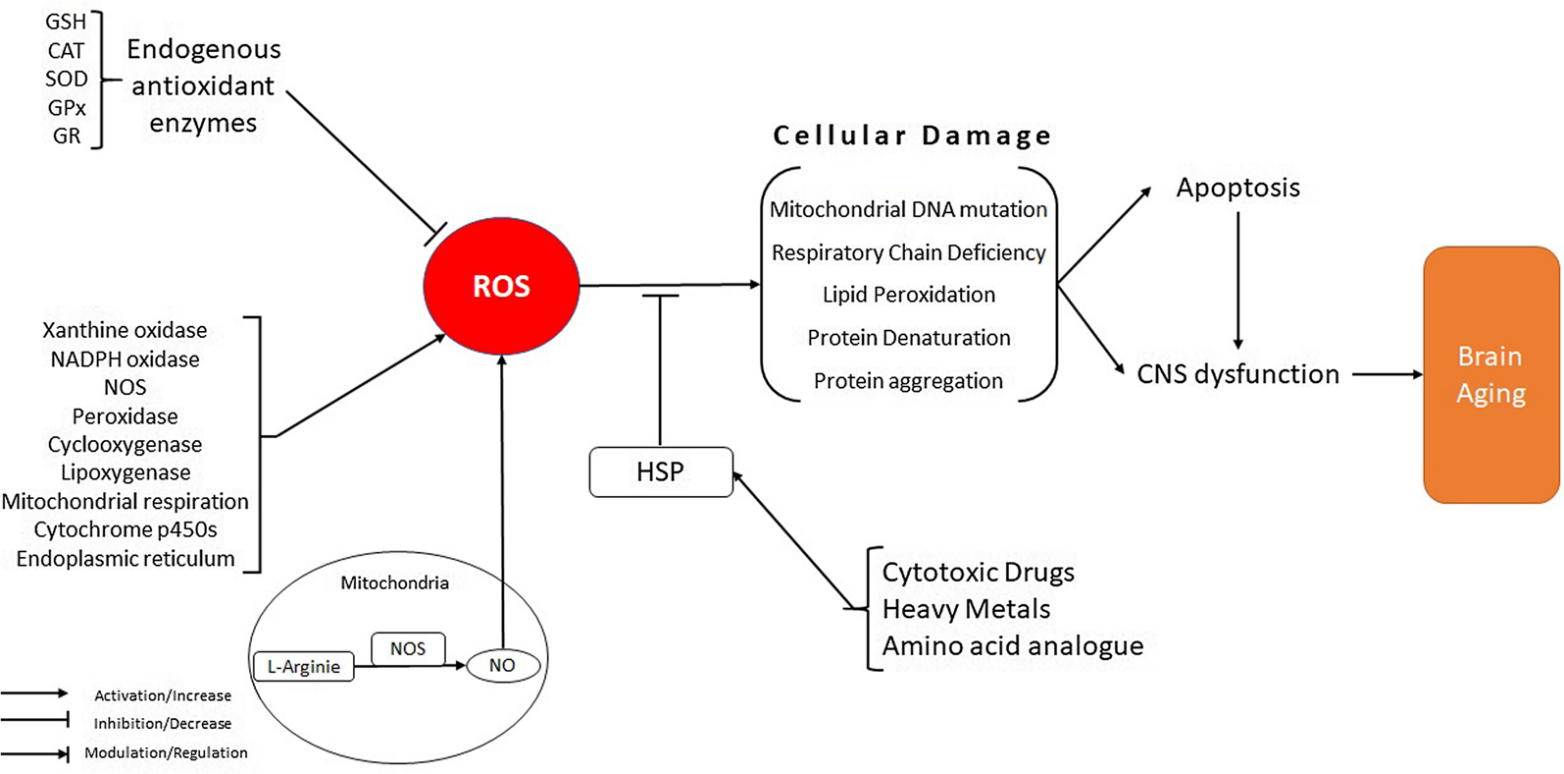
Role of oxidative stress in brain aging

Furthermore, enhanced generation of NO can lead to alterations in intracellular levels of GSH. Also, in cultures of astroglial cells, induction of iNOS declines total GSH, whereas increasing GSSG, and this effect was eliminated by pretreatment of glial cells with inhibitors of NOS [100]. In addition, an increased intracellular GSH before treatment of endothelial cells to NO donors almost eliminates activation of the HO-1 cascade, which exhibits that GSH can counteract NO via stimulating HO-1 [89]. Ultimately, these data support the idea that the decrease of antioxidant defense systems is as essential as ROS production in the injury of the aging brain (Fig. 1).

## Mitochondrial dysfunction and brain aging

Mitochondria are defined as the 'energy powerhouse of the cell' because they enable cells to produce more ATP than they could via oxidative phosphorylation. Mitochondria also provide biochemical energy for cellular function and are vital to our survival [101]. Mitochondria can grow larger, combine with other mitochondria, and divide. Also, dysfunctional mitochondria can be removed via lysosomes [102, 103].

Mitochondria have crucial roles in Ca^2+^ homeostasis within the cells and as the origin of signals that modulate transcription of the nuclear gene [104–106]. Also, it has been reported that mitochondria have a non-energetic role in the modulation of metabolism, apoptosis, and aging [107–109].

Most mitochondria genes are conveyed to the nuclear DNA (nDNA). The mtDNA encodes 13 subunits of oxidative phosphorylation. The remaining 76 subunits are encoded via nDNA made in the cytosol and then transferred to the mitochondria, which show functional interaction among both genomes [110, 111]. The functional communication between nuclear and mitochondrial DNA is essential for mitochondrial function and health. The lack of this interaction causes dysfunction of mitochondria and the decline of ATP synthesis [109].

**Fig. 2:**
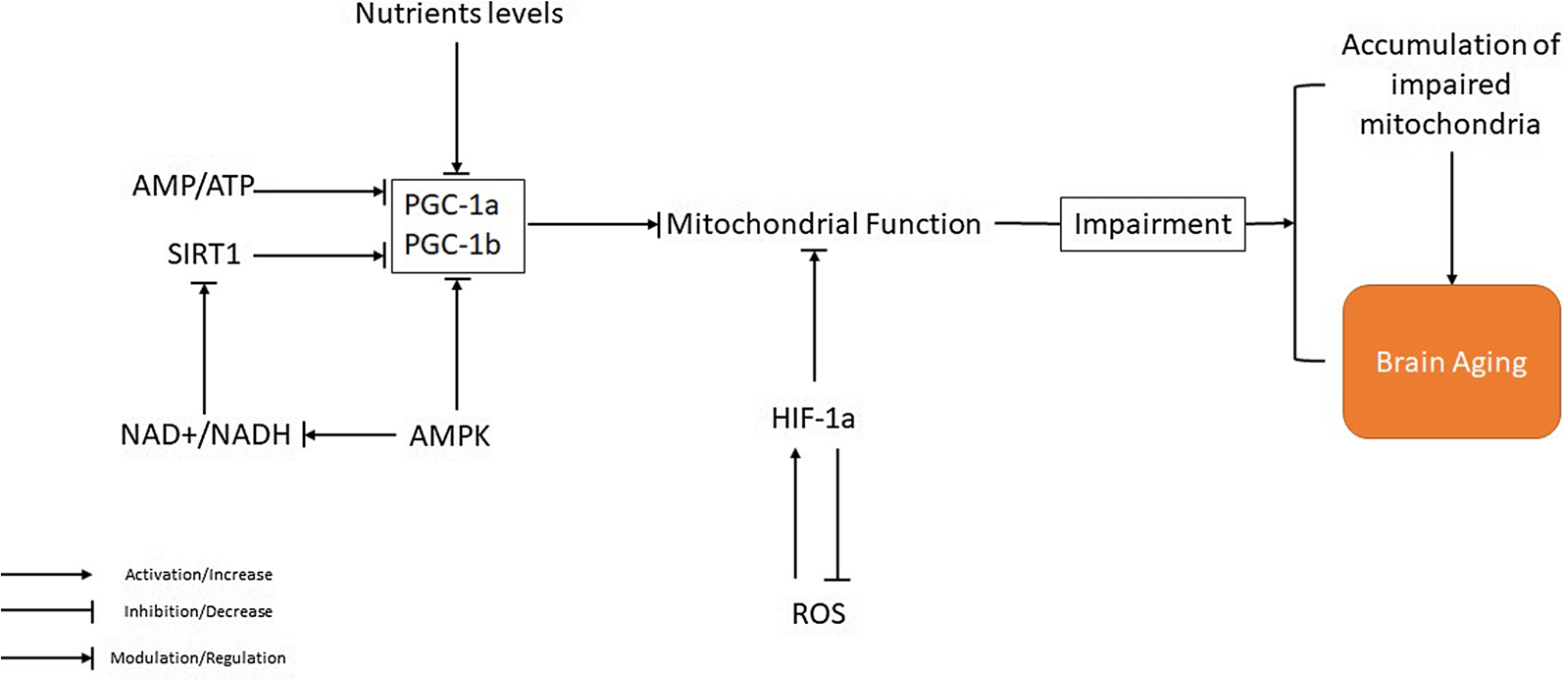
Role of mitochondrial dysfunction in brain aging

The regulation of mitochondria happens mostly via peroxisome proliferator-activated receptor gamma coactivator 1-alpha (PGC-1α) and PGC-1*β*, which respond to alterations in nutrient levels, like AMP/ATP and NAD + /NADH ratio (regulated through AMPK and SIRT1). The PGC-1*α*/*β* expression has an essential role in the biogenesis of mitochondria [112, 113]. Also, the mitochondrial function can be modulated via Hypoxia-inducible factor 1 alpha (HIF-1*α*). HIF-1α is recruited to mitochondria in response to oxidative stress. Mitochondrial HIF-1α maintains against oxidative stress-induced apoptosis. HIF-1α in mitochondria decreases ROS levels and reverses mitochondrial damage [114]. In the model organism *C. elegans*, the HIF-*α*-mediated ROS is the major lifespan determinant. However, the exact mechanisms involved are still not completely elucidated (Fig. 2) [115]. An animal study showed that calorie restriction or aerobic exercise similarly increased PGC-1α levels and spatial memory in mice. The combination of calorie restriction and aerobic exercise or physical exercise alone showed a higher increment in PGC-1α levels and spatial memory [116].

The brain's vulnerability towards mitochondrial dysfunction makes it a target of aging-induced progressive decline in mitochondrial function [117]. Mitochondria are spread across the axons and dendrites of neurons. They produce ATP that acts as a neurotransmitter in both central and peripheral nervous systems. ATP is also involved in chemical neurotransmission and also cellular maintenance and repair [118].

Axonal transport is essential for neurons' regular action and survival, transferring newly made substances from the soma to target cells across the axon and conveying trophic factors from synaptic terminals to the soma. Axonal transport is mediated via motor proteins that walk across microtubules. Fast axonal transport needs persistent energy through long distances to fuel the molecular motors that transport vesicles. The motors that are involved in fast axonal transport need ATP generated via mitochondria all across the axon. Therefore, impairment of mitochondrial ATP generation can disturb axonal transport, and disturbances in transport can intervene with mitochondrial trafficking, leading to axonal degeneration (Table 1).

**Table 1:** A summary of studies on the molecular and cellular pathways contributing to brain aging

Authors	Goals of Study	Study Subjects	Findings remarks
Coviello-McLaughlin and Prowse [150]	Determining relationship between telomere length and ageing	Mus spretus mice	Individual tissues had Independent regulation of telomerase activity and telomere lengths; Genetical control of telomere lengths; Gender differences in mean terminal restriction fragment length
Ain et al. [158]	Assessing telomere length and telomerase activity in non‐replicative and replicative neural brain populations	C57BL/6 mice	A cell cycle‐dependent and ‐independent manner in telomers lengths; No induction of telomerase activity and nuclear TERT protein by aging; Rela subunit of NF‐κB did not influence telomerase activity or telomere length; telomere instability was a failure to induce or relocate telomerase
Puhlmann et al. [160]	Assessing relationship between changes in leukocyte telomere length with structural plasticity (cortical thickness) of the brain and influence of mental training on telomere length	Healthy adults	Mental training intervention did not affect leukocyte telomere length; Association between short-term change in leukocyte telomere length and concomitant change in plasticity of the left precuneus extending to the posterior cingulate cortex
Gampawar et al. [159]	Investigating association between leukocyte telomere length with brain aging characteristics	Elderly individuals	Longer leukocyte telomere length was significantly associated with larger brain parenchymal fraction, larger white matter hyperintensities load and score; Leukocyte telomere length was associated with cognitive domain of attention/speed; Longer leukocyte telomere length protected the global brain volume and contributed to better cognitive functions, especially in the attention/speed domain in the elderly individuals
Yan et al. [225]	Assess whether Growth hormone (GH)/insulin-like growth factor-1 (IGF-1)-deficiency influences the outcome of cerebral ischemia induced by endothelin-1 with or without early-life growth hormone (GH) treatment	GH/IGF-1 deficient dw/dw rats	GH/IGF-1 deficiency did not affect infarct size but significantly attenuates brain edema and astrocytic infiltration compared with controls; GH-treatment reversed the cerebral ischemia deleterious effects
Pirger et al. [226]	Assessing effects of pituitary adenylate cyclase activating polypeptide (PACAP38) homolog on age-related complications	Aged Lymnaea	Exogenous PACAP38 promoted memory formation; Endogenous PACAP38 levels were low in the brain; Insulin-like growth factor-1 promoted memory formation
Rodríguez‐Muela et al. [396]	Investigating relationships between different lysosomal proteolytic systems in the retina during normal aging	Atg5flox/flox; nestin-Cre mice (a mouse model with downregulation of macroautophagy in neuronal precursors)	Increased chaperone mediated autophagy; reduced macroautophagic activity in the retina with age; Increased chaperone mediated autophagy in retinal neurodegeneration
De Biase et al. [397]	Assessing expression of autophagy markers and Amyloid precursor protein (APP) in aging	Old (aged 11–20 years) and young (aged 1–5 years) bovines’ brains	Impaired autophagy in aged bovine and not in the young animals; increased intraneuronal APP deposition with age
Park et al. [68]	Investigating involvement of superoxide-producing enzyme NADPH oxidase in aged-induced altered neurovascular regulation	C57BL/6 mice	Nox2 subunit of NADPH oxidase was a main producer of neurovascular oxidative stress and induced deleterious cerebrovascular effects with aging
Ulmann et al. [73]	Assessing the composition of fatty acids in brain and hippocampus in cognitive deficits	Aged and young Wistar rats	Decreased arachidonic acid concentration in aged rats; Significant phosphatidylserine and phosphatidylinositol classes in aged rats
Guebel and Torres [408]	Assessing the effects of sexual dimorphism on normal aging	Elderly-healthy individuals	Older women: Diminished expression of LC3, HDAC6, and PINK1; Older men: declined macroautophagic activity evidenced by increased expression of Bcl-2 (inhibitor of Beclin 1); Activation of mitophagy triggered by PARKIN evidenced by increased BAG-2 expression which inhibits PINK1 degradation
Head et al. [80]	Investigating the link between oxidative damage, aging and b-amyloid (Ab)	Canines brains	Increased lipid peroxidation; Increased oxidative damage of proteins (carbonyl formation); Age-dependent decline in GS activity and in the level of glutathione in the prefrontal cortex
Çakatay et al. [82]	Investigating the relation of oxidative protein damage parameters with oxidative stress in brain	Young, adult, and old Wistar rats	Protein carbonyl formation (oxidative protein damage parameter) was a marker of brain aging; Protein oxidation was related with lipid peroxidation in brain aging
Aksenova et al. [83]	Assessing aging and diet restriction’s effects on the brain	Male and female brown Norway rats	Diet restriction alleviated age-associated level of oxidative stress and reduced protein damage
Nicolle et al. [85]	Assessing protein and nucleic acid oxidative damage in the hippocampus in aging and impaired spatial learning	Long-Evans rats	Oxidative stress in aged hippocampal neurons evidenced by elevated heme oxygenase-1, elevated 8-hydroxy-2P-deoxyguanosine (an oxidative nucleic acid adducts); damaged mitochondrial DNA; Intensified oxidative stress in the learning-impaired rats
Paul et al. [87]	Investigating the roles of SOD2 in aging and associated pathologies	Sod2 mutants Drosophila	SOD2 protected against mitochondrial oxidative damage, neurodegeneration, age-related defects in behavior and early-onset mortality; SOD2 mutation resulted in mitochondrial aconitase activity, accelerated senescence of olfactory behavior, precocious neurodegeneration, DNA strand breakage in neurons, shortened life span, and increased age-dependent mortality
Lee et al.. [115]	Investigating effects of mild inhibition of mitochondrial respiration on extension of lifespan	Caenorhabditis elegans	Inhibition of mitochondrial respiration increased hypoxia-inducible factor HIF-1 (a transcription factor that activates genes responsible for promoting survival during hypoxia) activity in by elevating the level of reactive oxygen species in Caenorhabditis elegans
Ghosh et al. [134]	Assessing relation between oxidative redox shift with macromolecular damage in Alzheimer’s disease (AD)	3xTg-AD mouse model	Precedent of cognitive deficits in AD was a more oxidized redox state and a lower antioxidant GSH defense which both were dissociated from neuronal changes by ROS damage
Jaskelioff, Muller et al. [153]	Assessing whether reactivation of endogenous telomerase activity halts or reverses the degeneration in adult mice with severe telomere dysfunction	Mice with knock-in allele encoding a 4-hydroxytamoxifen (4-OHT)-inducible telomerase reverse transcriptase-Estrogen Receptor (TERT-ER)	Homozygous TERT-ER mice displayed short dysfunctional telomeres and sustain increased DNA damage signaling and classical degenerative phenotypes upon successive generational mating and advancing age; somatic telomerase reactivation reversed neurodegeneration with restoration of proliferating Sox^2+^ neural progenitors, DCX^+^ newborn neurons, and Olig^2+^ oligodendrocyte populations; telomerase-dependent neurogenesis alleviated hyposmia and recovered innate olfactory avoidance responses
Molofsky et al. [156]	Assessing whether genes associated with senescence functionally contribute to physiological declines in progenitor activity	Mice	Age-associated decline in progenitor proliferation in the subventricular zone and neurogenesis in the olfactory bulb which was partly related to p16INK4a (which encodes a cyclin-dependent kinase inhibitor linked to senescence); Age-declined multipotent progenitor frequency and self-renewal potential in forebrain
Bake et al. [224]	Investigating mechanisms underlying IGF-1’s neuroprotective actions un a post-stroke IGF-1 treatment animal model	Middle-aged female rats	IGF-1 downregulated disease-related miRNA in PI3K-Akt signaling, cell adhesion/ECM receptor pathways and T-and B-cell signaling; attenuated reduced phospho-Akt, reduced blood brain barrier permeability, and suppressed IL-6, IL-10 and TNF-a; suppressed IL-6 and reduced infarct volume by 39%
Cohen et al. [232]	Investigating relation between Insulin/IGF signaling pathway (IIS) and Alzheimer’s disease	AD model mice	Reduced IGF signaling was associated with protection from the Alzheimer's-like disease symptoms including reduced behavioral impairment, neuroinflammation, neuronal and synaptic loss; reduced IGF signaling caused sequestration of soluble Aβ oligomers into dense aggregates of lower toxicity
Demontis and Perrimon [383]	Investigating muscle functional decline in systemic aging	Drosophila	Progressive accumulation of protein aggregates in muscle aging was associate with impaired muscle function; FOXO and 4E-BP signaling delayed muscle functional decay, extended the lifespan, delayed age-related accumulation of protein aggregates outside muscle tissues via decreasing feeding behavior and the release of Insulin from producing cells
Zhang et al. [400]	Investigating the roles of 4-hydroxynonenal (HNE) in autophagy—lysosome pathway	Rat primary cortical neurons	HNE (lipid peroxidation product) affected autophagy through lysosome inhibition which resulted in accumulation of damaged and dysfunctional proteins and organelles and consequent neuronal death
Hara et al. [394]	Investigating the role of autophagy in neurodegeneration	Atg5 deficient mice	loss of autophagy caused accumulation of cytoplasmic inclusion bodies in neurons and resulted in progressive deficits in motor function and neurodegeneration even in the absence of any disease associated mutant proteins
Komatsu et al. [393]	Investigating roles of autophagy in neurons	Atg7 deficient mice	Impaired autophagy leaded to polyubiquitinated proteins accumulation in neurons as inclusion bodies, which increased in size and number with ageing; Impaired autophagy caused abnormal limb-clasping reflexes, reduced coordinated movement, death within 28 weeks of birth, and massive neuronal loss in the cerebral and cerebellar cortices
Fernández et al. [391]	Assessing effects of increased autophagy on mammalian lifespan	Mice with F121A (Becn1F121A/F121A) mutation in beclin 1 + mice deficient in Klotho3	Disrupted beclin 1/Bcl-2 complex disruption increased autophagy in F121A mutated mice; increased autophagy increased lifespan of F121A mutated mice and diminished age-related renal and cardiac pathological changes and spontaneous tumorigenesis; mice deficient in Klotho3 (an anti-aging protein) had increased beclin 1/Bcl-2 interaction, decreased autophagy, premature lethality and infertility which were attenuated by the beclin 1 F121A mutation
Chang et al. [389]	Assessing autophagic activity throughout life course	wild-type + long-lived daf-2/insulin/IGF-1 mutants + glp-1/Notch mutants Caenorhabditis elegans	age-related decline in autophagic activity in the intestine, body-wall muscle, pharynx, and neurons of wild-type animals; daf-2 and glp-1 long-lived mutants had regulated autophagy in distinct spatiotemporal-specific manners
Simonsen et al. [385]	Assessing the effects of increased autophagic gene expression in an aging nervous system	Drosophila	Enhanced Atg8a expression in older fly brains extended lifespan by 56% and promoted resistance to oxidative stress and the accumulation of ubiquitinated and oxidized proteins

Mitochondrial oxidative phosphorylation is the critical origin of energy supply and axonal transport in the cortical projection neurons (CPNs), which degenerate in AD [117]. Thus, these neurons are highly vulnerable to impairments in mitochondrial trafficking according to their extended dimensions. Respiratory chain enzymes and mtDNA are the main targets of mitochondrial damage [119]. The mitochondrial membrane permeability transition pores (mPTP) have been known as one of the primary regulators of mitochondria during apoptosis. The mPTPs are involved in brain development and pathologically in a range of age-related neurodegenerative diseases [120].

Mitochondria extracted from the brains of different animals exhibited several age-associated changes such as mitochondrial fragmentation or enlargement [121, 122], enhanced oxidative damage to mtDNA [123, 124], enhanced amounts of mitochondria with depolarized membranes [125], dysfunction of the mitochondrial respiratory chain [126–128], impaired Ca2 + handling [127, 129], and a diminished threshold for the formation of mPTP [130].

Decrement in the function of mitochondria through brain aging associates with a decrease in the levels of intracellular NAD + and the NAD: NADH ratio, which could be assumed to endanger the activities of the NAD + -dependent enzymes that are essential for neuronal viability and function, such as Sirtuins as histone deacetylases (HDACs) that require NAD for their enzymatic activity [131, 132]. Most cell lineages in the brain probably encounter the accumulation of impaired mitochondria through aging, as proposed from investigations of astrocytes and neurons identified from brains of young and aged mice [133, 134].

## Telomere and brain aging

Telomere shortening is one of the primary hallmarks of aging [9]. The telomere theory of aging and cellular senescence proposes that cells evaluate the number of divisions and define when replication is suitable [135]. Telomeres, also called the biological clock, consist of thousands of tandem DNA repeats, TTAGGG, at the end of each linear chromosome. The telomeres have a crucial role in genome maintenance and promote stability through replication procedure, preventing chromosomal end fusion and unnecessary recombination [136, 137]. In somatic cells, telomeres shorten gradually after each cell division [138]. Therefore, a restricted number of cellular divisions are expected, which cells lead to replicative senesce [139].

Like stem cells, telomere shortening can be reversed via the enzyme known as telomerase in some cell types. Telomerase is a ribonucleoprotein (RNP) enzyme that consists of two essential subunits: the telomerase reverse transcriptase protein (TERT) and the telomerase RNA (TER) [140]. The ability of embryonic stem cells (ESCs) and also, induced pluripotent stem cells (iPSC) to divide limitlessly is according to the up-regulation of telomerase enzyme in these cell populations [141, 142].

The role of telomere shortening and its involvement in the healthy aging procedure of the brain and neuron senescence at the cellular level is not entirely illustrated [143]. In addition, age-associated alterations, particularly in neurons, are still understudied. Because neurons are post-mitotic, cell division as the main factor for telomere erosion has been considered absent in neurons once they reach terminal replication. This opinion has been disputed by finding DNA content variations, obviously demonstrating a cell cycle activity in around 10–20% of post-mitotic neurons, as shown for the cortex of healthy aging brains and AD [144, 145].

Studies have shown that inducing telomerase activity in somatic cells restores numerous aging features, such as senescence [146]. In somatic tissues, including the CNS, telomerase enzyme exhibits deficient transcript levels and activity, which vary in their relation with protein levels, e.g., in the murine cortex [147–149]. The telomerase function in post-mitotic cells like neurons is mainly unassociated with telomere elongation but somewhat related to cell survival-promoting function. Interestingly, Eitan et al. reported a dissociation between the decreased telomerase activity through postnatal development and the persistent levels of TERT mRNA and proteins [149]. Interestingly, in another study, Coviello-McLaughlin et al. was reported that telomeres in the brain might not pursue the telomere theory of replicative senescence and aging. They showed that in the brain of *M. spretus*, a mouse strain with telomeres with the same length as in humans, telomere shortening is not related to telomerase enzyme and cell turnover activity and even with the more substantial slope in comparison to other organs with limited cell turnover. This was found postnatally, whiles telomere shortening through aging was not found [150]. However, during the first few days of stem cell differentiation, the newly produced neurons are susceptible to apoptosis triggered by telomere damage [151].

Recent investigations have demonstrated that hippocampal TERT is involved in modulating mood behaviors by regulating the proliferation of neural progenitor cells (NPCs) and is required for spatial memory formation. In this regard, hippocampal-dependent learning and memory functions and neurogenesis in the hippocampus are decreased in telomerase-knockout mice [152]. In confirming, Telomerase enzyme reactivation in telomerase-knockout mice returns olfactory neurogenesis to normal status by a subsequent improvement of an olfactory dysfunction [153].

The cumulative data from more recent investigations suggest that the features of cellular senescence are not limited to replication-competent cells. In the CNS, the Post-mitotic neurons obtain a senescence-like feature due to DNA damage and telomeres dysfunction [154]. When cells go through senescence, they terminate dividing, produce pro-inflammatory cytokines, resistant to apoptosis, and express the proteins p16Ink4a and p21 [155]. The cellular senescence might be the fate of some NPCs through aging, as demonstrated by up-regulation of p16Ink4a linked with decreased amounts of proliferating NPCs in the sub-ventricular zone (SVZ) [156]. Furthermore, when human NPCs are kept in culture, they show a restricted amount of cell divisions and then go through cellular senescence [157].

Ain et al. reported that telomere attrition happens in the aging mice brain. It is not limited to neural populations with regular cell cycle activity (e.g., in glia). Ain et al. found that both cell cycle-independent and -dependent alterations in telomere length happen in the aging brain. They also provided evidence that suggests non-replicative neurons involved in the age-related telomere erosion procedure detected in the brain [158]. However, there are controversial reports describing leukocyte telomere length (LTL) associated with structural alterations to the brain and decreased cognitive capacity during aging [159, 160].

Structural brain indices are hallmarks of individual differences in aging and health [161]. Gampawar et al. reported a highly unique link between LTL and brain parenchymal fraction (BPF) in the elderly. Also, they found that Longer LTL is connected with a larger brain and with better cognitive functioning in the attention/speed mediated by BPF [159]. In another study by Puhlmann et al., short-term LTL change was connected with structural brain alteration [160]. Collectively, future investigations in which senescent cells are eliminated from the aging brain of the model organisms should illustrate whether cellular senescence is a true hallmark of aging in the brain.

## Inflammation and brain aging

The immune system is one of the most pivotal protective physiological systems of the organism [162]. Aging affects the function of the immune system, which is termed immunosenescence [163, 164]. The participation of senescent cells in host immunity is associated with the release of pro-inflammatory cytokines. This phenomenon is defined as senescence-associated secretory phenotype (SASP). Given the pro-inflammatory nature of SASP, cellular senescence in different organs and tissues remarkably promotes inflammation in the elderly [165].

SASP is stimulated basically via NF-κB in response to oncogenic stress and DNA damage, which initiates the transcription of a host of genes including tumor necrosis factor-*α* (TNF-*α*), interleukin-6 (IL-6), IL-8, IL-1β [166, 167]. NF-*κ*B is a transcription factor induced via inflammatory mediators and ROS that contributes to the deleterious and protective responses, depending on the types of induction that lead to co-activation of different cascades. NF-*κ*B also induces genes that regulate cellular survival, differentiation, inflammation, growth, and cell death [168, 169]. If there is no stimulation, NF-κB is bound by the inhibitor of κB (IκB), preventing nuclear translocation. Also, direct oxidation of NF-κB by ROS inhibits its DNA binding ability.

Moderate amounts of ROS result in the phosphorylation and degradation of IκB, enabling the NF-κB activation [170, 171]. After NF-κB activation, it plays a pro-survival role via suppressing c-Jun N-terminal kinases (JNKs) and caspase-mediated programmed cell death, up-regulating the anti-apoptotic genes and proteins involved in declining mtROS, particularly the genes, encoding antioxidant manganese-type superoxide dismutase (MnSOD) [172]. On the other hand, high concentrations of ROS activate Nf-kB via protein kinases. This Nf-kB activation plays a central role in stress signaling pathways including, JNK, extracellular signal regulatory kinases (ERKs), mitogen-activated protein kinase (MAPK), Src family kinases (SFK), Akt, and phosphatidylinositol-4,5 bisphosphate 3-kinase (PI3K) [173].

Similar to other organs, inflammation is a typical characteristic of brain aging [174]. It has been shown that the age-induced increase of pro-inflammatory markers (CRP, IL-6, IL-1β, TNF-α) is associated with cognitive decline [165, 175]. A systematic review of 13 randomized clinical trials showed that doing physical exercise (aerobic and resistance) reduces inflammatory markers in healthy adults of all ages. Interestingly, older adults who performed high-intensity aerobic exercise benefited more than other ages in reduced inflammation [176]. In the same concept, a randomized study investigated the effects of aerobic, resistance, and neuromotor exercises in adults older than 60 years. It was shown that adults who performed these exercises had a significant reduction in TNF-α and IL-6, increased BDNF, and improved executive functions and attention [177]. The underlying mechanisms for the anti-inflammatory effects of physical exercise are thought to be due to the release of muscle-derived anti-inflammatory substances in older adults [178].

Microglia are the brain resident macrophages providing its innate immune defense. Microglia, a kind of glial cell, arise from erythro-myeloid precursors in the yolk sac, which inter the CNS during development [179, 180]. Microglia have dual roles in the nervous system. In the healthy adult brain, microglia are ramified cells that exhibit highly motile processes, which constantly survey brain parenchyma in response to harmful agents, neuronal cell injury, or infections. By releasing trophic factors and regulator cytokines, microglia are involved in neuroprotection [181–183].

On the other hand, neuroinflammation is characterized by microglia. It is described by enhanced amounts of an intricate set of mediators, such as TNF*α*, TGFβ, and IL1β, which are enhanced in elderly individuals. When neurons are damaged as a consequence of neurodegeneration or aging, microglia are activated via the production of neurotransmitters, ATP, cytokines, and ion changes in the local environment [179, 180, 184]. Links between neuroinflammatory activation of microglia and neuronal loss and decreased neurobehavioral function and cognitive impairment have been shown. Neuroinflammation causes intricate communication with oxidizing agents via redox sensors localized in receptors, transcription factors, and enzymes. These factors affect the connection between neurons and glia and neuronal function, resulting in neurodegenerative changes [185, 186]. Also, after activation, microglial cells express a stimulable form of NOS and generate high levels of NO that lead to oxidative damage to neurons.

Furthermore, toll-like receptors (TLRs), critical for inducing innate immune responses to invading pathogen, are involved in neuroinflammation in age-associated brain diseases [187]. Hence, microglial TLR4 receptor activation can aggravate neural degeneration in models of age-associated neurodegenerative diseases. In contrast, repression of microglial activation has been defined as a therapeutic method to mitigate microglia-based neuroinflammation in cerebral ischemia [188].

Irregular activation of immune cells leads to functional impairment and degeneration of synapses in neurodegenerative diseases and brain aging; when correctly regulated, the same cascades play crucial roles in neuronal stress resistance and neuroplasticity. For instance, TNF-α plays a pivotal role in learning, memory, and synaptic plasticity in the hippocampus [189]. Also, TLRs play pivotal roles in innate immunity and modulating neural plasticity [187]. In mice, hippocampal neurons that are deficient in TNF-α receptor show enhanced susceptibility to degeneration and dysfunction in models of AD, TBI, and epileptic seizures [190–192] and also TLRs 2 and 4 modulate energy intake and metabolism and help regulate critical aspects of the autonomic nervous system (ANS) [187].

Astrocytes might also involve in adaptive responses to age-associated neuronal stress. These cells eliminate glutamate from synapses, generate neurotrophic factors, and improve bioenergetic activity in neuronal cells. These astrocyte functions might be impaired through aging, thus aggravating pathological neuroinflammatory procedures [193–195]. The activation of NF-κB by TNFα is necessary for neuronal survival, which protects cells against β-amyloid (Aβ) neurotoxicity. Further, NF-κB stimulates anti-apoptotic responses and maintains neurons from ischemic and excitotoxicity brain damage [196–199]. Moreover, NF-κB activation has a pivotal role in the initiation and perpetuation of inflammation via its response to TNF*α*- mediated inflammatory stimuli, resulting in the stimulation of numerous chemokines and cytokines. Furthermore, NF-κB and MAPK activation are essential in oxidative stress and Aβ-induced neuronal cell death [200–202].

**Fig. 3:**
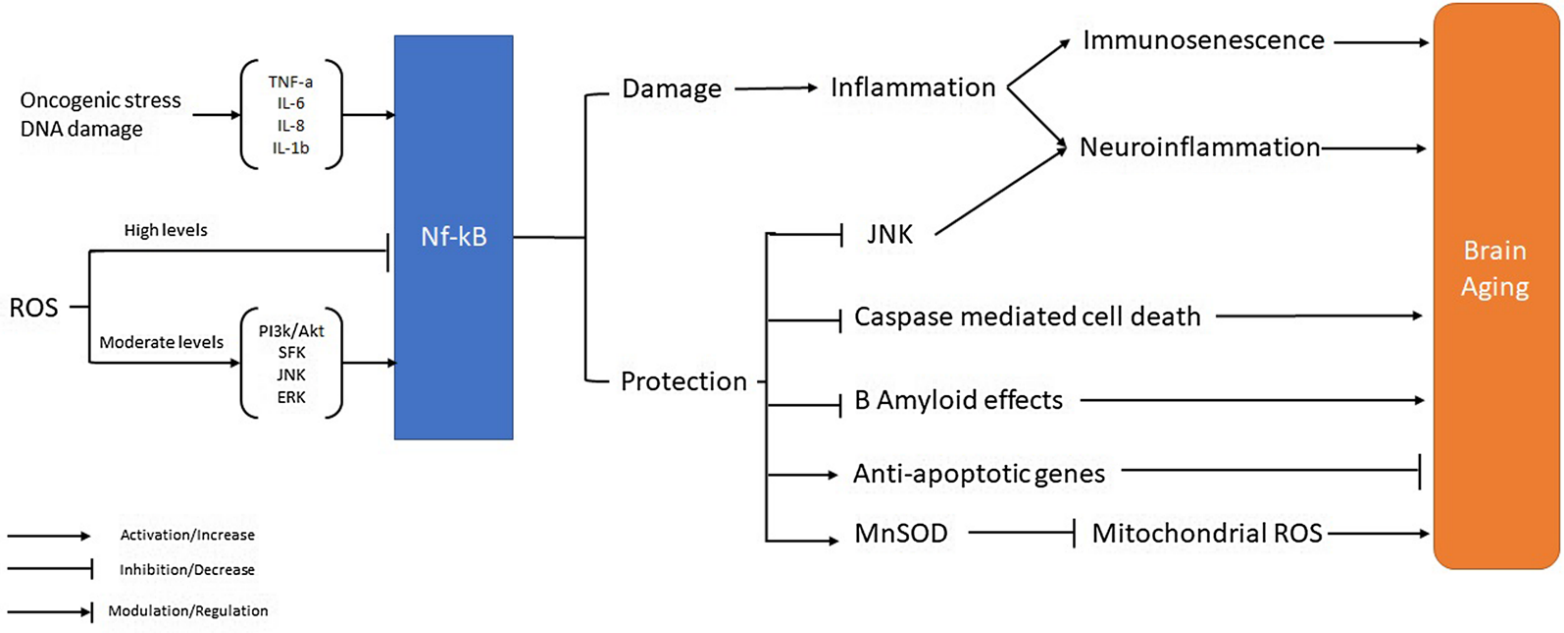
Role of inflammation in brain aging

Besides NF-κB, various transcription factors are induced via inflammatory responses, like signal transducer and activator of transcription (STAT-1) and peroxisome proliferator-activated receptor-gamma (PPARγ), which also have been involved in AD (Fig. 3), [203, 204].

## Metabolic control and brain aging

As age advances, the supply of cellular energy decreases. The responsible biochemical pathways of this decline have an essential role in longevity and healthy aging. Different evolutionary conserved signaling pathways and transcriptional modulators, like insulin/ insulin-like growth factor 1 (IGF1) signaling (IIS), the mammalian target of rapamycin complex (mTORC), AMP-activated protein kinase (AMPK), and Sirtuins pathways regulates the metabolic function during aging [205, 206]. Also, factors like ROS and p53 signaling seem to be part of the central metabolic pathways in living cells [207, 208].

The IIS pathway is one of the well-known signaling pathways in longevity conserved from yeast to mammals [209]. The IIS pathway regulates proliferation, survival, and metabolic processes. Insulin and IGF1 bind to the insulin receptor (IR). Signaling adapter proteins, like insulin receptor substrate (IRS1-4) proteins, bind to tyrosine residues, which activate AKT and PI3K and act upon various target pathways like mTOR GSK3β [210, 211].

Over the last decade or more, it has been clarified that mutation in some parts of the insulin/PI3K cascade remarkably influences longevity [210, 212]. Both the cerebral vasculature and CNS are essential targets for IGF-1 and GH [213, 214]. During aging, secretion of GH and subsequently hepatic generation of IGF-1 decline remarkably. This IGF-1 decline contributes both to cerebrovascular disorders and age-associated cognitive impairment [215–217]. The available data propose that the deficiency of GH/IGF-1 promotes atherosclerosis development and also cerebral hemorrhage during aging [218–220]. The intensity of cerebral damage following acute ischemia was shown to enhance through aging, leading to deficiency of IGF-1-mediated neuroprotection [221–223]. Also, recent investigations describe that post-stroke intracerebroventricular (ICV) administration of IGF-1 to aged rats remarkably declines infarct volume [224]. Yan et al. [225] reported GH/IGF-1 deficiency in the Lewis dwarf rat, as the aging model did not remarkably change the infarct size after endothelin-1-induced focal cerebral ischemia. In a study by Pilger et al. [226] in pond snail (*Lymnaea stagnalis*), a novel invertebrate model that was broadly used for the study of learning and memory reported that *L. stagnalis* had age-associated impairment of memory, which could be restored upon IGF-1 treatment [226]. Neuroprotective effects of IGF-1 through mammalian aging have been reported [213, 214, 227]. Likewise, IGF-1 deficiency has been engaged in the pathogenesis of several age-associated disorders and modulation of longevity in mammals [213, 214, 228]. Thus, neuroprotective and anti-aging effects of IGF-1 in *L. stagnalis* can be related to mammalian aging and providing more validation of the model. Low levels of IGF-1 have been found in humans who had remarkable longevity [229]. During 12 h of chronic restraint stress for eight weeks, mice were divided into either access to running wheels or no access. Mice with access to physical exercise by running wheels showed reduced chronic restraint stress-induced cognitive impairment and improved cell proliferation in the dentate gyrus, possibly via increase of IGF-1 and increased activity of glutathione s-transferases (GST) [230].

**Fig. 4:**
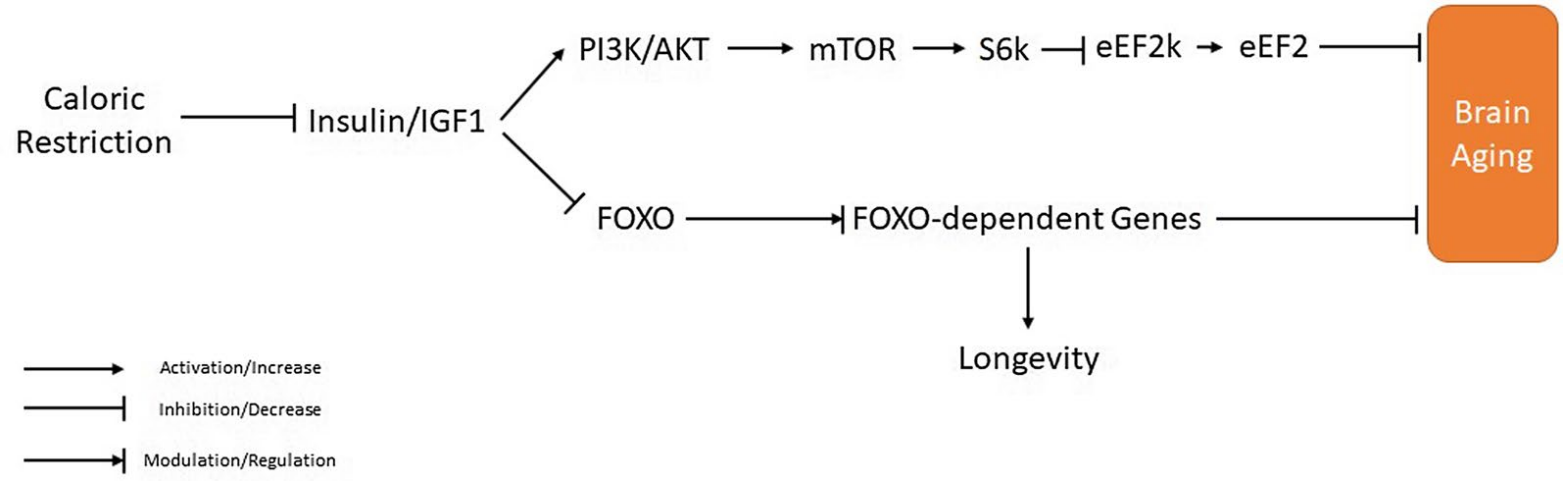
Molecular pathway involved in caloric restriction effect on brain aging

Some conflicts exist between the neuroprotective effects of the IIS pathway and its adverse effects on longevity. Interestingly, knockout of IRS2 receptor can decrease cognitive impairment and neurodegeneration in mouse models of AD [231, 232]. Furthermore, in patients with AD, downregulation of the IIS pathway was reported [233]. Therefore, the IIS pathway as an effective neuroprotector and an indicator of the neurodegenerative procedure is disputable. IGF-1 induces an intracellular signaling cascade mediated via the PI3K-AKT pathway, enabling the phosphorylation of FoxO transcription factors (Fig. 4) [234]. FOX (forkhead box) proteins regulate lifespan in some of the simple invertebrate organisms, including *C. elegans* [235–237]. The "O" subclass of the FOX family (FoxO) consists of evolutionarily conserved isoforms that in mammals include FoxO1, FoxO3, FoxO4, and FoxO6 *C. elegans* DAF-16 and DFoxO in *D. melanogaster*. FoxO proteins' activity is associated with different cellular processes, including cell differentiation, glucose metabolism, cellular detoxification, DNA repair, and apoptosis [235–237]. The insulin/IGF‐1 pathway initiates intracellular signaling mediated by AKT, enabling phosphorylation of three conserved residues within the FoxO transcription factors. The phosphorylation of FoxO by AKT results in the export of proteins to the cytosol. It suppresses FoxO-dependent genes' expression (234, 235); however, in the presence of cellular stress or the lack of growth factor signaling, FoxOs transfer into the nucleus and result in the expression of FoxO‐dependent genes. Numerous mechanisms of how FoxO proteins promote the longevity of model organisms have been proposed. In model organism *C. elegans*, mutations in Age-1, a homolog of PI3K, extend the lifespan in model organisms. Also, mutations in Daf-2, a homolog of IR, extend lifespan in *C. elegans* [238, 239]. Also, it has been reported that the loss of CHICO as an IRS homolog enhances longevity in *D. melanogaster* [240]. The typical role of Daf-2 is to counteract the activity of Daf-16 as the homolog of the FoxO transcription factor. At the same time, Daf-2 mutation increases resistance to stress and lifespan via activation of Daf-16. In fact, in the *daf-2*^*−/−*^ strains, AKT-1-mediated inhibition of DAF-16 is down-regulated and enabling DAF-16 translocation to the nucleus to enhance the expression of target genes. Direct target genes of DAF-16/FoxO are responsible for the stress resistance and longevity phenotypes connected with *daf-2*/IR mutations [241, 242].

The protein kinase mTOR is a serine/threonine kinase that applies its functions as a catalytic subunit of two distinct protein complexes, mTORC1 and mTORC2 [243]. The mTOR pathway contributes to the development and aging process in living cells by regulating cellular growth and metabolic procedures in response to nutrients and nutrient-induced signals, like insulin [244]. mTOR pathway activates ribosomal protein S6 kinase (S6K), which in turn phosphorylates eukaryotic translation elongation factor 2 kinase (eEF2K) at Ser366, leading to its inhibition and thus promote dephosphorylation and activation of eEF2, which modulates gene transcription and protein synthesis [245, 246]. The mTOR pathway is downstream of the insulin/PI3K signaling cascade and acts as the primary determinant of longevity [244]. Decreased activation of the PI3K/AKT/mTOR signaling pathways remarkably extends longevity in mice [247]. Also, centenarians that represent enhanced susceptibility to insulin have declined mTOR activity. Therefore, lifespan appears to be linked with the declined activity of the insulin/IGF-induced PI3K/AKT/mTOR signaling cascades [244, 248].

Moreover, S6K directly phosphorylates IRS proteins on multiple serine residues, consequently inhibit the insulin pathway [249]. Also, cellular exposure to compounds like rapamycin postpones cellular senescence. It decreases mitochondrial dysfunction via the inhibition of the mTOR pathway [250].

AMPK is one of the primary regulators of cellular energy levels [251]. When cellular energy levels have been compromised in mammals, this pathway is activated by increases in ADP/ATP and AMP/ ATP ratios. In this condition, the AMPK response stimulates alternative pathways that produce ATP besides inhibiting ATP consumption [251, 252]. Therefore, AMPK activates a set of compensatory pathways, including stimulation of glucose uptake, inhibition of fatty acid synthesis, fatty acid oxidation (*β*-oxidation), and enhanced mitochondrial biogenesis [253]. At the molecular level, some compounds such as metformin inhibit the mitochondrial respiratory chain in the liver and increase the levels of AMPK, which is beneficial in improving insulin sensitivity, lowering cAMP, thus reducing the expression of the gluconeogenic enzyme and also increasing physical performance and longevity [254]. Additionally, the activation of AMPK enhances longevity and is associated with metabolic improvement in mice [10]. Although, how AMPK acts on aging is very intricate and still requires more investigations to be illustrated.

The Sirtuins are evolutionarily conserved from bacteria to mammals. These protein complexes play a crucial role in regulating metabolism, insulin resistance, energy supply, oxidative stress, inflammation, neuroprotection, and longevity [255, 256]. In mammals, there are different subtypes of Sirtuins that are located in various cellular compartments, including mitochondria (SIRT3, SIRT4, and SIRT5), cytosol (SIRT2), and nucleus (SIRT1, SIRT6, and SIRT7)[257]. The Sirtuins family of proteins is categorized as class III histone deacetylases that transfer acetyl group to ADP-ribose of NAD^+^ generating a 2′-*O*-acetyl-ADP-ribose, nicotinamide, and deacetylated protein. Both SIRT1 and SIRT2 genes are up-regulated in the brain [258, 259]. Also, SIRT1 directly interacts with the PIK3 adaptor subunit p85 and constructing a complex that, upon insulin induction, binds to IRS1/2 and stimulates this signaling cascade. Furthermore, levels of SIRT1 are associated with the phosphorylation of AKT at serine 473. Other targets of SIRT1 proteins are AMPK, IRS, glutamate dehydrogenase (GDH), histones, acetyl CoA synthetase, and proteins involved in regulating stress responses, metabolic pathways, and cell survival [258–260].

**Fig. 5:**
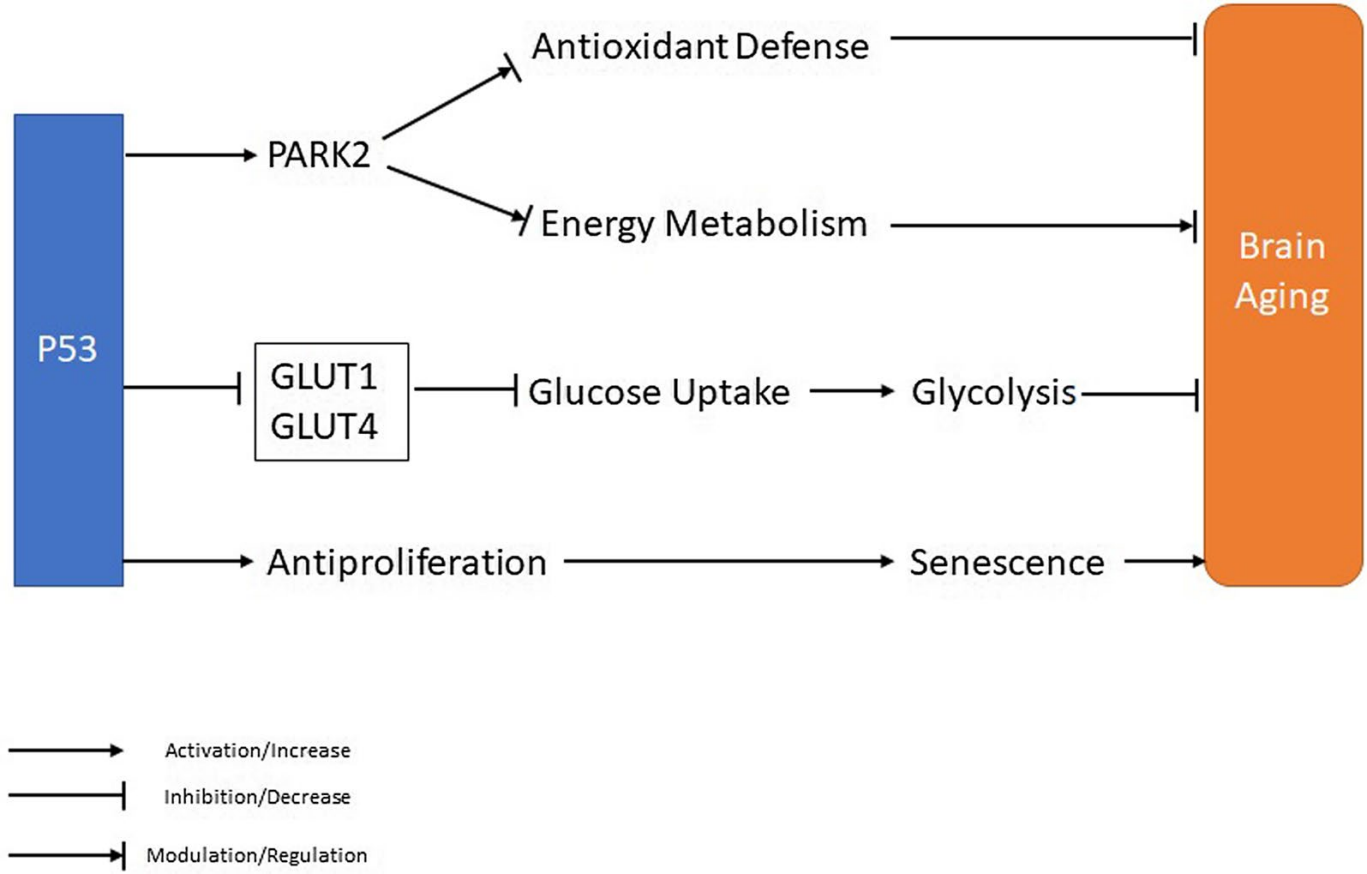
Role of p53 protein acts on the cellular energy supply during the process of aging

The p53, as tumor suppressor protein, can stimulate a series of anti-proliferative procedures, like cell cycle arrest, resulting in cellular senescence and apoptosis at the presence of cellular stress [261]. Furthermore, p53 plays an essential role in regulating and monitoring cellular energy levels and modulating some pathways like oxidative phosphorylation, glycolysis, insulin sensitivity, *β*-oxidation, mitochondrial integrity, and autophagy [262, 263]. Glucose transporters (GLUTs) promote glucose transport through the cellular plasma membrane, which is the first rate-determining stage in glucose metabolism. Experiments have been shown that p53 directly inhibits glycolysis via suppressing the transcription of GLUT1 and GLUT4 to decrease glucose uptake [263, 264]. p53 also indirectly suppresses GLUT3 expression via down-regulation of the activity of NF-κB, which up-regulates the transcription of GLUT3 in the cells. p53 also prevents the GLUT1 translocation to the plasma membrane to inhibit glucose uptake. One of the primary mechanisms by which p53 prevents the translocation of GLUT1 to the plasma membrane is via up-regulation of its target RRAD, which binds to p65 of the NF-κB and prevents NF-κB activity from inhibiting GLUT1 translocation [264, 265]. p53 regulates a broad spectrum of proteins involved in glycolysis, acting as a modulator of glycolytic activity [266, 267]. Moreover, p53 promotes oxidative phosphorylation via transcriptionally increases the cytochrome c oxidase assembly two protein (*SCO2*) expression, which is required for the assembly of the catalytic core of cytochrome c oxidase (COX) and apoptosis-inducing factor (*AIF*). p53 suppresses the transcription of pyruvate dehydrogenase kinase-2 (*PDK2*), which is a negative modulator of pyruvate dehydrogenase (PDH). This enhances the conversion of pyruvate into acetyl-CoA for entry into the tricarboxylic acid (TCA) cycle, which positively modulates glucose oxidation. p53 also stimulates the Parkin (*PARK2*) expression, a component of an E3 ubiquitin ligase complex and modulator of antioxidant defense and energy metabolism [268, 269]. Therefore, p53 protein modulates aging acts via linking the cellular energy supply with the stage of senescence progression (Fig. 5) [270, 271].

AKT is involved in many physiological processes in the central and peripheral nervous system, including the regulation of neuron survival [272]. Also, the PI3K-AKT signaling pathway is a critical modulatory step in the response of the cells to oxidative stress. During brain development, the PI3K pathway is involved in various cellular functions, such as cell migration, proliferation, and axon guidance [273]. Furthermore, the activity of PI3K is necessary for the transport of microtubule during neuronal polarization as well as neuronal growth and formation [274]. Also, PI3K/Akt pathway through cAMP-response element-binding (CREB) protein induces fibroblast growth factor 2 (FGF2) and activates FGF receptor 1 (FGFR1). PI3K/Akt modulates neural hippocampal progenitor proliferation and differentiation through FGF2 and FGFR1 [275].

CREB is a cellular transcription factor that modulates the expression of essential genes in dopaminergic neurons and molecules essential for long-term memory and neuronal plasticity [276, 277]. Dopamine acts exclusively through G-protein-coupled receptors (GPCRs), which affects the phosphorylation of CREB via its GPCRs [278]. Brain-derived neurotrophic factor (BDNF) is highly conserved in gene function and structure through vertebrates between the different neurotrophins. It serves as a significant regulator during brain development synaptic plasticity. The CREB plays a crucial role in mediating neurotrophin responses in neurons since the exposure of neurons to BDNF induces CREB phosphorylation and activation, which acts as an essential regulator of BDNF-induced gene expression (279). Also, it has been found that the CREB itself can be regulated by BDNF [276, 279]. Animal studies have shown a positive association between CREB mRNA and BDNF with memory improvements[280].

One of the growth factors contributing to the beneficial effects of physical exercise is BDNF.

Animal studies have shown that voluntary exercise increases BNDF expression and production in the hippocampus, cerebellum, and frontal cortex. Inhibition of BDNF activity suppressed the physical exercise-induced improvement of cognitive test performance [281]. It has been shown that BDNF availability has a direct correlation with cell proliferation in the hippocampus [281]. BDNF also affects neurotransmitter release and synaptic plasticity [282, 283]. Furthermore, the effect of physical exercise on neurotransmitters can be through BDNF as it regulates neurotransmitters, including dopaminergic and cholinergic systems. It has been shown that reduced intrinsic availability of BDNF by gene deletion or inhibition impairs LTP [284, 285]. Accordingly, abolishing BDNF by exogenous administration restored the ability to induce LTP in the hippocampus [286].

It was shown that following a 3-week running period, mice had elevated BDNF levels compared with sedentarism. Elevated BDNF levels persisted until two weeks after the running period. However, BDNF levels returned to pre-exercise levels three weeks after the running period. Furthermore, elevated BDNF levels were correlated with cognitive improvement in the radial water maze [287]. In the same context, it was shown that aerobic physical exercise in non-demented sedentary older adults aged between 55 and 80 years was associated with gained volume in the anterior hippocampus and improved spatial memory. Also, an increase in levels of BDNF was seen with the gain of volume in the anterior hippocampus [39]. Accordingly, clinical studies have shown that some specific polymorphisms in the BDNF gene can induce learning impairments in individuals [288]. Clinical studies have shown that physical exercise affects BDNF production in brain regions responsible for the cognitive activity, such as the hippocampus and caudal cortex [289]. Overall, these findings show that physical exercise can protect against age-induced impairments in cognitive activity by regulating BDNF. In the concept of AD, it has been shown that animal models of AD have BNDF deficits which can be upregulated by physical exercise and an enriched environment [290–292].

In contrast, in an AD transgenic mice model, physical exercise was associated with decreased BDNF. The possible explanation for these conflicting findings is not comparable to AD animal models and types of physical exercise. Various mechanisms have been suggested for physical exercise-induction of BDNF activity in the brain, including neurotransmitters with their receptors and peripheral factors such as estrogen, corticosterone, and possibly insulin-like growth factor 1 (IGF-1) [40, 41, 293–297]. Physical exercise can affect neurogenesis by regulating the systemic production of IGF-1. It was reported that physical exercise-induced neurogenesis was abolished by blocking the entrance of IGF-1 to the brain [293]. In addition, IGF-1 facilitates the physical exercise induction of angiogenesis as systemic administration of IGF-1 increased angiogenesis. Its inhibition reduced the formation of new blood vessels in the brain of animals. The underlying mechanisms for IGF-1-induced angiogenesis are thought to be through induction of VEGF by IGF-1. Accordingly, it was shown that IGF-1 suppression reduced the secretion of VEGF and hence the formation of new capillaries [298]. Studies have shown that physical exercise increases the levels of IGF-1 and VEGF both in young and old aged individuals [299]. Also, IGF-1 acts as an upstream regulator of BDNF expression as peripheral IGF-1 administration increases the BDNF expression in the brain [293, 295, 300]. Physical exercise was shown to increase acetylcholine (Ach) levels and muscarinic receptor expression in the hippocampus of adult rats [297]. Studies have shown that physical exercise can regulate BDNF expression in the hippocampus through direct modulation of Ach [294, 301]. In confirmation, recombinant human BDNF induced the activity of basal forebrain cholinergic neurons and dopamine uptake in vitro [302].

Studies have shown that environmental enrichment increases BDNF levels, especially in the hippocampus [303]. Similarly, a fivefold increase in BDNF levels was found after one year of environmental enrichment [304]. In confirmation, BDNF deletion led to impairments in LTP and synaptogenesis following mental activity [286, 305]. In an animal model of Huntington's disease, BDNF levels were increased in the striatum following exposure to environmental enrichment. Moreover, animals showed improved survival and motor functions [306].

Also, CREB is one of the primary mediators of the PI3K/Akt pathway. There is likely crosstalk among Akt/CREB pathway and susceptibility genes [307]. Moreover, Early investigations have shown that up-regulation of AKT in neurons inhibits apoptosis through growth factor withdrawal [308]. AKT1 and AKT2 expression in CNS are enhanced in the early steps of embryonic development but decline gradually in post-natal cells [309]. In the adult brain, the AKT1 and AKT2 expression are weak.

In contrast, a significant enhancement in the AKT1 expression is stimulated when cells are exposed to a harmful stimulus [310, 311]]. The expression of AKT has demonstrated to be essential and adequate for the survival of neurons, as a dominant-negative mutant of AKT, or the suppression of PI3K, that result in programmed cell death of neurons even in the presence of neurotrophic factors and stimulates apoptosis through oxidative stress [312, 313]. Moreover, investigations have shown that intracellular AKT protects cells against damage. In different paradigms of neuroprotection, phosphorylation of AKT modulates neuronal survival elicited by antioxidants [312–314].

It has been shown that physical exercise enhances adult neurogenesis in the dentate gyrus and increases synaptic plasticity. In this regard, the PI3K activation promotes the survival of adult-born neurons in the dentate gyrus granule cells produced through physical exercise, which consequently enhance synaptic plasticity [315].

The PI3K/AKT signaling pathway has an essential role in synaptic plasticity, learning, and memory procedure in the brain. PI3K/Akt pathway is involved in the maintenance and induction of long-term potentiation (LTP) in the hippocampal CA1 region of adult brains [316]. The PI3K/Akt pathway has been proposed for LTP at the perforant path-granule cell synapses. ICV infusions of LY294002 as an inhibitor of PI3K pathway impaired maintenance of LTP in the hippocampus and are linked with phosphorylation of AKT at Ser473 [317]. Furthermore, PI3K/Akt pathway has been reported to be needed for amygdala fear conditioning [318]. Accordingly, after infusion of the LY294002 together with wortmannin as inhibitors of PI3K and also rapamycin as an inhibitor of mTOR pathway, into the medial prefrontal cortex (mPFC) impaired the long-term retention of trace fear memories [274].

The PI3K‐Akt pathway is also required for the receptor trafficking at the synaptic membrane, affecting synaptic plasticity as part of neuronal function and development in the CNS [319, 320]. AKT pathway is also implicated in the regulation of synaptic strength through GABA receptor phosphorylation. Moreover, the induction of insulin stimulates the PI3K activation at dendritic synapses, which then stimulates AKT activation in the post-synaptic space [321].

It has been shown that exposure to BDNF or neurotrophin 3 (NT-3) promote maturation of developing neuromuscular synapses, also axonal and dendritic sprouting, and alterations in the number of synapses in hippocampal neurons, which PI3K pathway promotes long-lasting synaptic plasticity that depends on levels of protein synthesis in both pre-and post-synaptic vicinities mediated through the induction of the eukaryotic translation initiation factor-alpha (eIF2-α), that is the main target of mTOR pathway [322, 323]. Also, oxidative stress linked with elevated cholesterol uptake impairs insulin signaling, enhances serine phosphorylation of IRS1, and inhibits insulin-induced PI3K and AKT activities, resulting in enhanced stress-induced JNK activity that increases cognitive impairments in mice [324].

Observational, preclinical, and clinical trials provide evidence that CR can increase the lifespan by 1–5 years while improving health and quality of life [325]. CR modulates the intrinsic processes of aging by adapting the cellular and metabolic mechanisms. The mild cellular stress following CR stimulates the survival-promoting mechanisms [326, 327]. This has been shown as CR exhibited protection against brain aging and neurodegeneration by increased neurotrophic factors, particularly BDNF and GDNF [328–333].

Another element of the survival mechanisms induced by CR against aging is chaperone proteins. Chaperone proteins promote the correct folding of functional proteins and eliminate non-specific aggregation of non-native proteins. Chaperons also regulate apoptosis by modulating apoptosis inducers such as caspases [334–336]. It has been shown that CR protects against neurodegeneration by increasing the HSP family of chaperons, especially heat-shock protein-70 (HSP-70) and glucose-regulated protein 78 (GRP-78), in several brain regions of aging rodents [337–341]. It has been suggested that CR causes neuroprotection by both limiting the inflammatory response of microglial cells and also stimulating the neuroprotective phenotype of microglia by inducing the production of interferon-γ (INF-γ) [342, 343]. Calorie restriction reduced insulin and glucose levels while increasing glucose and insulin sensitivity in rodents and monkeys [344–347].

Furthermore, calorie restriction increased glucose utilization in rats by suppressing TNF-α mediated insulin resistance through negative regulation of NF-κB [348, 349]. CR decreases insulin and IGF-1 levels in plasma, resulting in decreased PI3K/Akt pathway activity. Decreased activity of PI3K/Akt reduces the phosphorylation of FoxO transcription factors. Thus the translocation of FoxO from the nucleus decreases. This results in decreased cytoplasmic FoxO levels and increased expression of FoxO factors. Consequently, the expression of FoxO regulated genes increases [350–353]. According to lifespan increasing effects of CR, increased expression of FoxO factors has positive correlations with increased lifespan in worms and flies [354, 355].

Peroxisome proliferator-activated receptors (PPARs) and their co-factors as PPAR coactivator 1 (PGC-1) regulate genes involved in response to stressful stimuli regarding nutrients and their metabolism [349]. Expression of PPARs has been shown to decrease in aging and be counteracted by CR in primates [356, 357].

Limited food sources upregulate the homolog of the Sirtuins family of proteins in yeast named the silent information regulator 2 (SIR2). SIR2 gene was first found in the yeast and acted as the silencer of the extra copies of mating-type information [358, 359]. SIR2 overexpression prolonged lifespan in yeast, worms, and flies[360–362] and shortened lifespan when inactivated [363]. It has been shown that in the brain of mice exposed to calorie restriction, SIRT1 (the mammalian ortholog of SIR2) and NAD levels are increased [364]. In line with this, SIRT1 overexpression decreased the Aβ peptide by increasing the cleavage of amyloid precursor through α-secretase in primary neuronal cultures [364]. Also, some studies have shown that SIRT1 may decrease the toxicity of Aβ by modulating the activity of the NF-κB transcription factor [365, 366]. Furthermore, SIRT1 homolog, SIR2, has shown neuroprotective effects against Huntington's disease by recovering dysfunctional neuronal phenotype in a nematode model of the disease [367]. Taken together, CR has shown protection against brain aging and neurodegenerative diseases by modulating BDNF and GDNF, HSP family of chaperon proteins, microglial cell activity, insulin and glucose pathway, FoxO transcription factors, PPARs family of nuclear hormone receptors and, Sirtuins family of proteins.

## Autophagy and brain aging

Autophagy or "self-eating" relates to the degradation procedure of damaged organelles, protein aggregates, and toxic substances that are entrapped within the lysosome [368]. Autophagy is necessary to maintain cellular homeostasis and integrity by providing metabolites needed to survive cells under extreme stress conditions [369]. Furthermore, autophagy can help maintain cellular energy status through nutrient restrictions during the catabolic pathway, which functions as a fueling and recycling procedure to provide vital energy and building blocks for the synthesis of the macromolecules [370]. There are different kinds of autophagy: microautophagy, macroautophagy, and chaperone-mediated autophagy (CMA). All of these are various in their functions and mechanisms of action [371]. Microautophagy is the non-selective lysosomal degradative procedure, which includes direct engulfment of cytosolic cargo at a boundary membrane via autophagic tubes, which mediate invagination of vacuolar membrane and scission of vesicle into the lumen [372]. Macroautophagy, commonly known as autophagy, needs to form a double-membrane structure called the autophagosome, which enclose cellular material and later fuse with the lysosomes [373]. CMA ensures the selective degradation of cytosolic proteins. Substrate proteins bind to the lysosomal membrane through the specific receptor called lysosome-associated membrane protein type 2A (LAMP-2A) [374].

Between the different signaling networks modulating autophagy, two kinases are associated with lifespan and aging regulation, including mTOR and AMPK [375]. mTOR, a negative modulator of autophagy, combines signals from growth factors and nutrient pathways to regulate cellular metabolism and growth [376]. Under nutrient-rich growth conditions, mTOR inhibits autophagy via direct phosphorylation of unc-51-like autophagy, activating kinase 1 (ULK1) and ATG13, which sequesters the ULK1 complex in an inactive state. Conversely, extreme stress conditions, like nutrient deficiency, inhibit the mTOR pathway and enhance autophagy [377]. However, in nutrient-deficient conditions, AMPK is activated and promotes autophagy by activating ULK1 via direct phosphorylation of Ser 317 and Ser 777. Also, AMPK stimulates autophagy by inhibiting the mTOR pathway via phosphorylating Raptor. This adaptor protein is essential for mTOR kinase activity. Ultimately, AMPK induces tuberous sclerosis complex 1/2 (TSC 1/2), inhibiting mTOR [375, 377, 378].

Autophagy can maintain cellular homeostasis when faced with different stress during aging, such as growth factor withdrawal, nutrient deprivation, damaged proteins and organelles, and genotoxic stress [379]. The recycling functions of autophagy reduce these stresses by removing impaired organelles and other damaged cellular components. Therefore, stress-mediated autophagy is part of a broader metabolic shift that promotes cells and organisms' survival by prioritizing maintenance and repair overgrowth [380]. Aging is associated with decreased autophagy in different model organisms. Early investigations exhibited that aged rats, *C.elegans,* and human cells have diminished lysosomal degradation compared to younger counterparts [381, 382]. Also, aging is associated with the down-regulation of numerous *ATGs* in *D. melanogaster* and rodent tissues [383–385]. In humans, the expression of *ATG5*, *ATG7,* and *BECN1* is decreased through normal aging. At the same time, age-related disorders like osteoarthritis, cardiomyopathy, and neurodegeneration show declined autophagy [386–388]. Recent work in *C. elegans* establishes a significant decrease in autophagic flux in vivo through the lifetime, noted by an age-related aggregation of immature autophagosomes and diminished autophagic degradation in all tissues examined [389]. Moreover, the aggregation of unremoved autophagosomes through aging might worsen neuronal dysfunction in age-associated disorders in worms [390]. In mice, an age-related decline in the amounts of autophagosome has been found, as may be anticipated by the age-associated decrease in *ATG* expression in humans [391].

Because neurons are usually considered post-mitotic cells and must protect their intricate function and structure integrated into neuronal networks during the organism's lifespan, the capability to eliminate dysfunctional and damaged molecules and organelles is an essential function survival of neurons. Saha et al. reported an age-related lack of macroautophagy function in dopaminergic neurons in the *C. elegans* model organisms [392]. Also, deletion of the Atg7 gene in mice results in neuronal loss and death within 28 weeks [393]. similarly, the absence of the Atg5 gene causes progressive deficits in motor function that are in line with the accumulation of cytoplasmic inclusion bodies in the mouse brain [394]. Further, amounts of Beclin 1 in the brain were reported to be decreased in old naked mole-rats, proposing an age-related decline in macroautophagy in this species [395]. Also, Rodríguez-Muela et al. [396] investigated age-associated alterations in autophagic mechanisms in the mouse retina. They reported lower Beclin 1 and LC3-II after the lysosomal blockage in older mice. However, De Biase et al. [397] reported lower Beclin 1, but an enhance in the LC3-II/LC3-I ratio in old cow brain specimens.

Lysosome pH gradients are primarily maintained via the plasma membrane V-ATPase, which pumps protons into the lysosomal lumen by consuming ATP [398]. Impairment of V-ATPase influences lysosomal acidification, which disturbs the removal of substrates and results in various diseases, such as neurodegenerative disorders [399]. Also, HNE can impair the function of lysosomes in cerebral cortical neurons that leads to the accumulation of cargos that are not degraded and consequent cell death [400]. Furthermore, the most remarkable morphologic alteration in neurons through normal aging is the accumulation of autophagic vacuoles filled with lipofuscin or neuromelanin pigments [401].

Autophagy plays a substantial role in removing damaged mitochondria which are known as mitophagy [402]. The accumulation of damaged and dysfunctional mitochondria in dopaminergic neurons is characteristic of PD. Also, defective mitophagy contributes to mitochondrial dysfunction in PD [403]. PINK1 and Parkin have been involved in suppressing inflammatory responses triggered via mitochondrial dysfunction, which leads to the loss of dopaminergic neurons in PD [404, 405]. Although knockout of *Pink1* and *Prkn* in mice failed to promote PD-related phenotypes, and similarly in mice and *D. melanogaster*, Pink1/Parkin was not essential for mitophagy. These findings suggest that other factors modulate mitophagy and PD [406, 407].

Guebel and Torres 

**Fig. 6:**
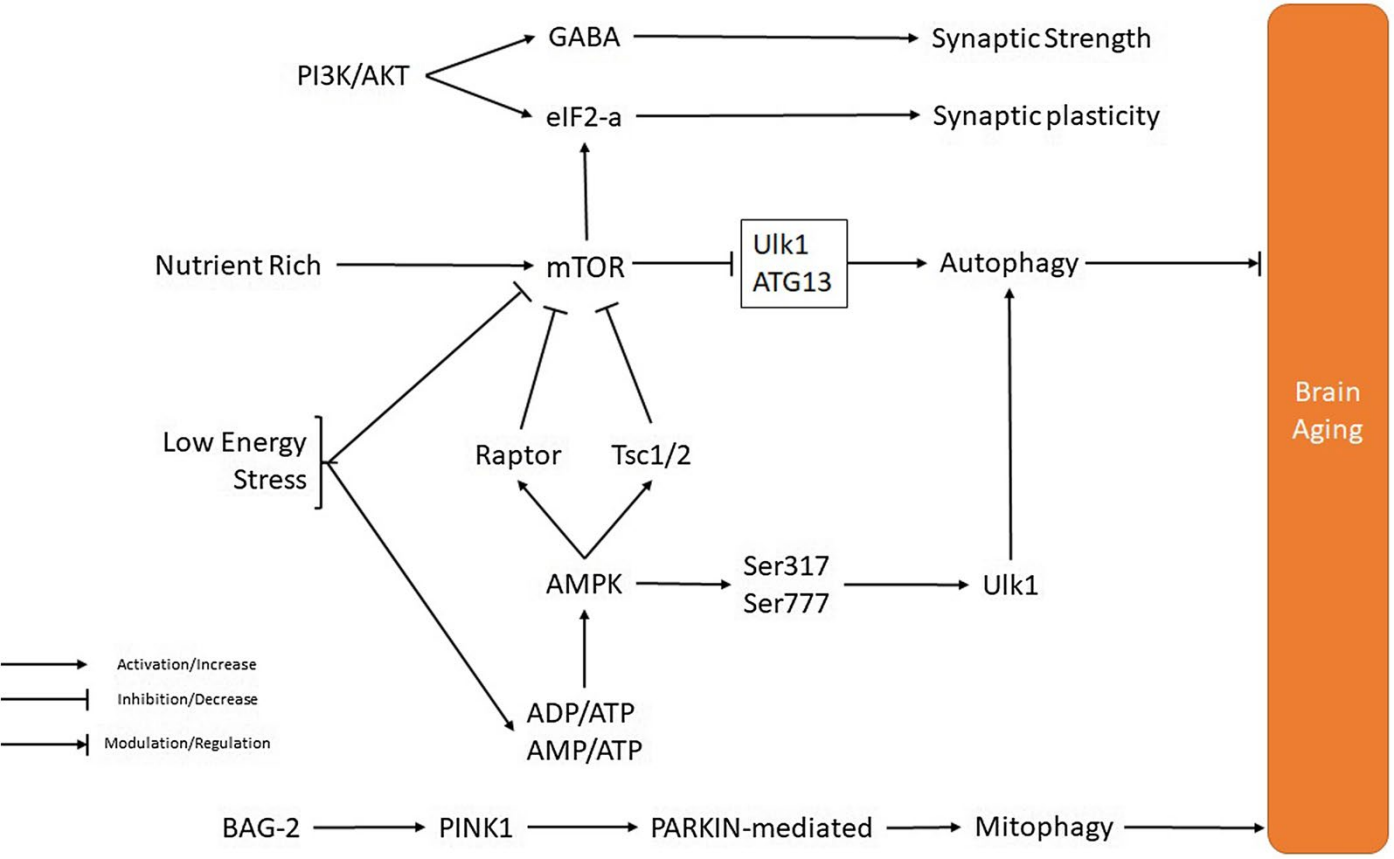
Role of autophagy pathway in brain aging

[408] investigated the effects of gender and aging on gene expression in the hippocampus. They reported diminished expression of LC3, HDAC6, and PINK1 in older women. Enhanced expression of Bcl-2, an inhibitor of Beclin 1, in older men proposed a decline in macroautophagy activity. Inversely, enhanced expression of BAG-2 expression, which inhibits PINK1 degradation via suppressing its ubiquitination and triggers PARKIN-induced mitophagy, in older men proposed activation of mitophagy. These investigations emphasize the intricate role of autophagy in the homeostasis of mitochondria. Also, there is a need for more investigation to reveal how mitophagy acts to prevent age-associated neurodegeneration. Thus, autophagy appears to be a compensatory response to cellular metabolic status, either to acquire energy or to remove impaired mitochondria. Finding the interrelationship between both modulators is necessary to link the energetic signaling to metabolism. Figure 6 indicates an association between the autophagy pathway and brain aging.

## Conclusion

Aging is an intricate and multifaceted biological procedure that includes all organ systems. Cellular dysfunction and accumulation of senescent cells are frequent in the central part of them. As well as, investigation in the areas of aging and neurobiology has demonstrated various factors during brain aging at the molecular, cellular, and tissue levels. Aging is the leading risk factor for a broad range of neurodegenerative disorders. Brain diseases of aging have recently become the main reasons for disability and death, according to advances in the treatment and prevention of some challenging diseases such as cardiovascular disease and cancers, which have enabled more people to live over 70. Gene expression profiling and brain imaging techniques provide a powerful tool to identify global alterations at the molecular, cellular, and behavioral statuses in aging brains. After having an overview of the recent developments on brain aging, in the present review, it has been described as the result of declined neurogenesis and synaptic plasticity, altered neurochemical and signaling pathways, including increased oxidative stress, impaired mitochondrial function, glial cell activation, impaired protein processing, and neuroinflammation.

Moreover, the neocortex and hippocampus are the most vulnerable areas, affected in aging with a variable degree of molecular and cellular alterations in their sub-centers. Although each of these age-associated alterations is common in the process of normal aging, their combined influence, together with the genetic background and environmental conditions, could trigger the solemn circle of cytotoxic activation. These alterations are associated with transcription factors, proteins, and environmental factors within the cell, like redox potential. Although, the main factor regulating the whole procedure remains uncertain. One of the first candidates would be to find how the redox capacity defines gene transcription and promotes responses in metabolism during brain aging.

Moreover, animal models have demonstrated that metabolic control and calorie restriction (CR) can significantly affect brain aging and susceptibility to neurodegenerative disorders. Recent findings suggest that calorie restriction and physical exercise increase neuroplasticity and resistance of the brain to stress and aging. Finding the cross-talk between the main pathways and their genetic and environmental regulation will be the main challenge in brain aging and neurodegenerative diseases.

Our understanding of brain aging is still at the beginning. More investigation is needed to establish effective therapeutic methods and drug development against brain aging. Furthermore, non-pharmacological methods, such as physical exercise, lifestyle changes, and calorie restriction, could enhance healthy aging via their effects on promoting the brain's physiological functions while decreasing ROS production and inflammation. Understanding the mechanisms that underlie the hallmarks will be essential to designing future interventions to halt or even reverse brain aging. Collectively, the primary purpose of investigation in neurobiology and brain aging should be to determine methods and strategies to promote successful brain aging in all people.

## Data Availability

The data that support the findings of this study are available in the body of the text.
